# Supplementary material for: Collective exchange processes reveal an active site proton cage in bacteriorhodopsin
Source: Commun Biol. 2020 Jan 3;3:4. doi: 10.1038/s42003-019-0733-7 (PMC6941954; doi:10.1038/s42003-019-0733-7)
Supplement: Supplementary file 1 — Supplementary Information [file 42003_2019_733_MOESM1_ESM.pdf]

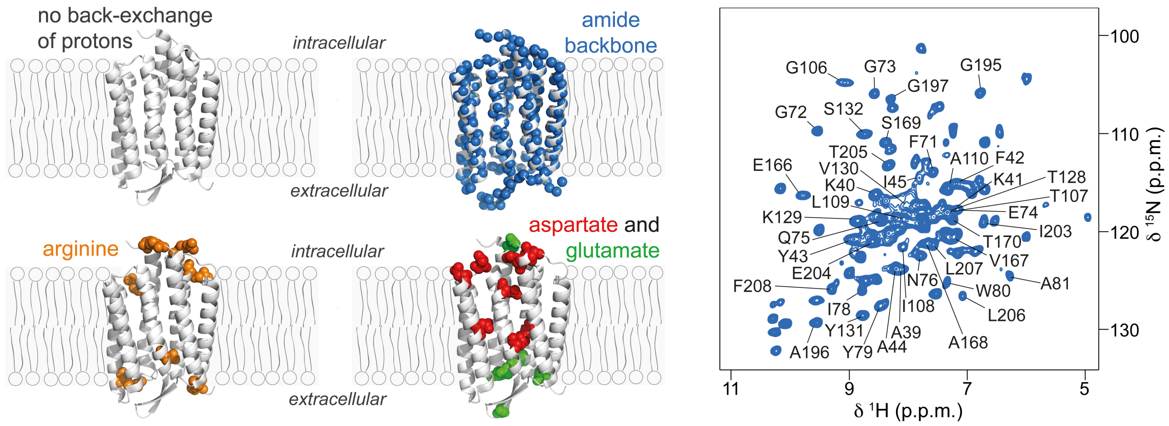

Supplementary Figure 1. Accessibility of protons in bacteriorhodopsin (BR, pdb 1c3w) by proton-detected magic angle spinning NMR. After introducing protons into the sample during illumination, signals of the backbone amides (blue), arginine side chains (orange) and carboxyl group protons in aspartic and glutamic acids (red and green, respectively) in the core of the protein can be expected. The proton-detected, two-dimensional ^{15}N-^{1}H correlation spectrum of BR in the purple membrane yields resonances of the amides in the protein backbone. Signals that could be unambiguously assigned are labeled with the respective amino acid.

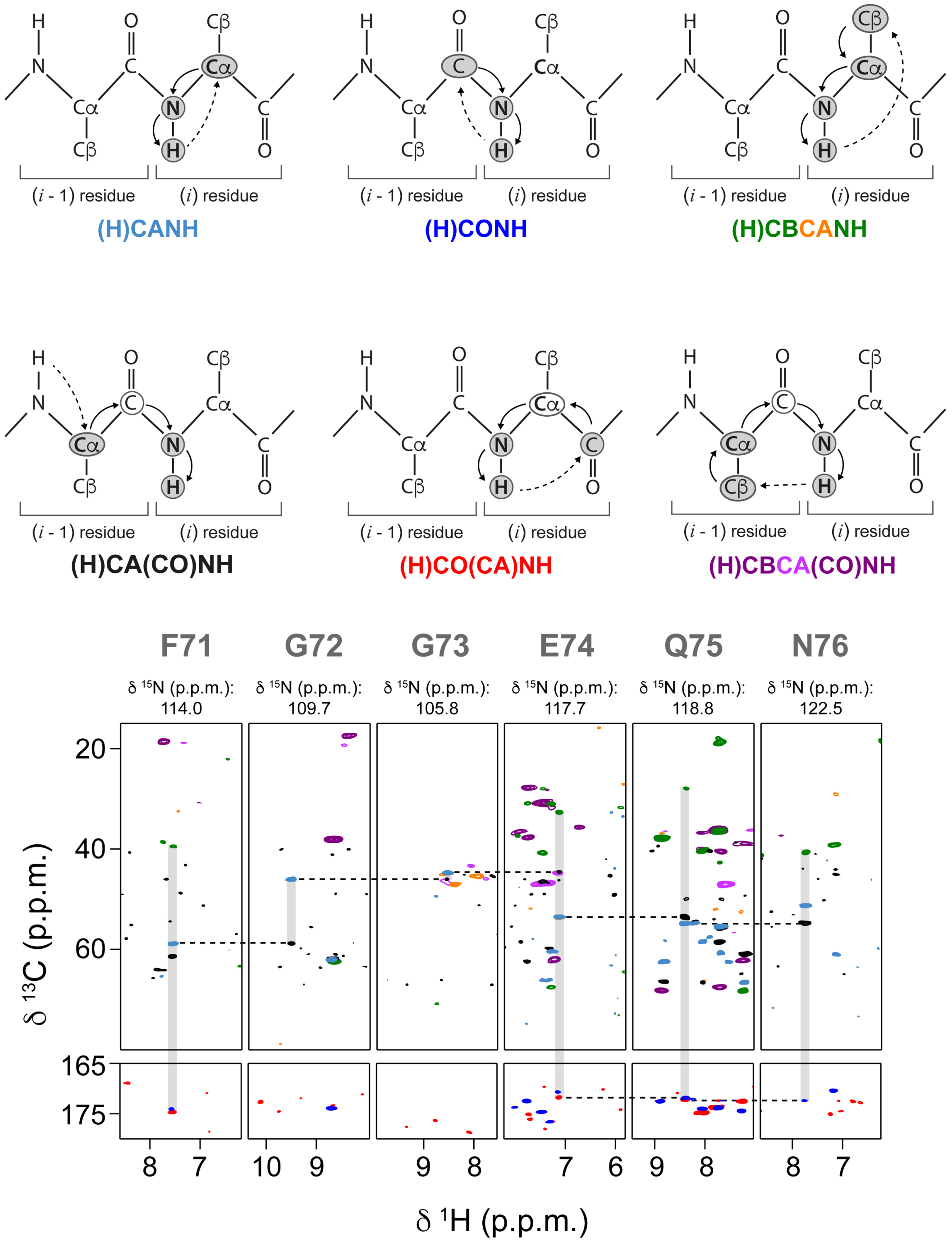

Supplementary Figure 2. Assignment of amide backbone signals in bacteriorhodopsin using triple-resonance magic angle spinning NMR experiments. As an example, sequential connections in two-dimensional strips of triple-resonance, three-dimensional (H)CANH (light blue), (H)CA(CO)NH (black), (H)CONH (dark blue), (H)CO(CA)NH (red), (H)CBCANH (positive contours (CA) in orange, negative contours (CB) in green) and (H)CBCA(CO)NH (positive contours (CA) in magenta, negative contours (CB) in purple) spectra are shown for residues F71 to N76. Magnetization transfer pathways of the six experiments are schematically illustrated at the top. Dashed lines indicate correlations allowing the assignment.

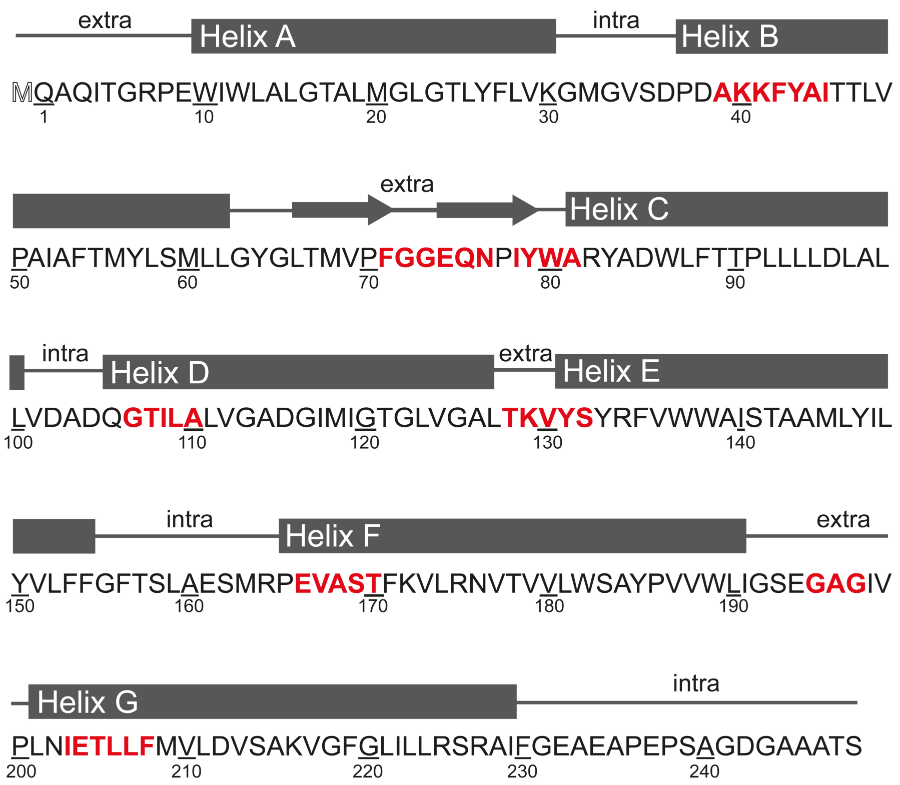

Supplementary Figure 3. Secondary structure of bacteriorhodopsin and assigned backbone signals. Black boxes indicate transmembrane α-helices, black arrows β-strands and black lines represent loops or unstructured protein regions. 'Extra' and 'intra' refer to extracellular and intracellular regions, respectively. Residues with assigned amide backbone signals are highlighted in red.

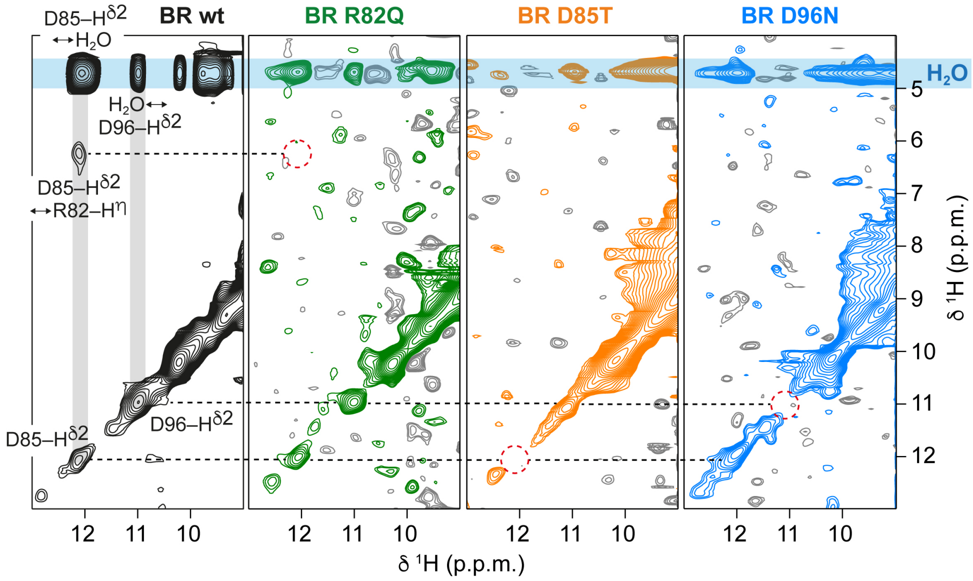

Supplementary Figure 4. ^{1}H-^{1}H exchange spectra of BR wildtype (wt, black spectrum), R82Q (green spectrum), D85T (orange spectrum) and D96N (blue spectrum). Negative contours are depicted in grey. The shown spectra are the same as in Fig. 2d, but the three mutant spectra are plotted at lower contours to visualize the noise level. This illustrates that the D85–$H^{\delta2}$, D96-$H^{\delta2}$ and R82–H^{η} protons are missing in the mutants, even when taking different signal-to-noise ratios into consideration.

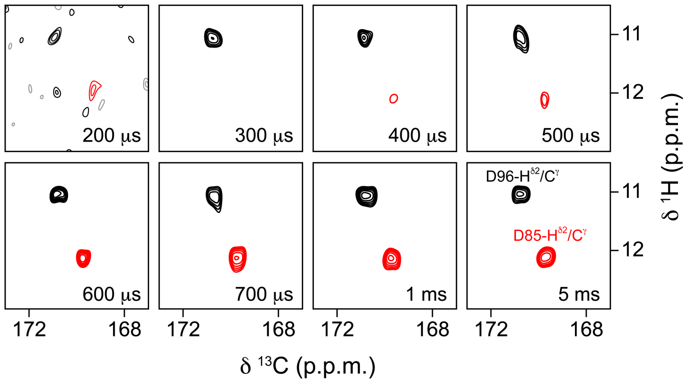

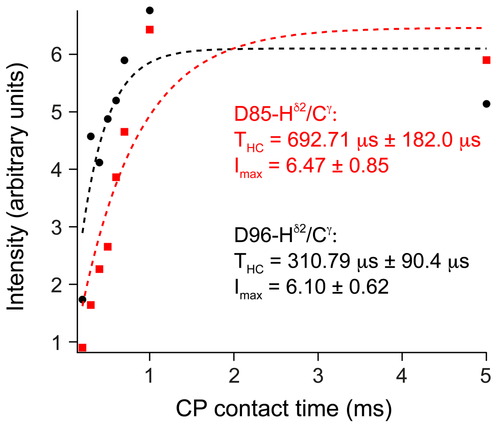

Supplementary Figure 5. Cross polarization (CP) build-up experiments of the D85 and D96 carboxyl group proton cross peaks. Eight dipolar coupling-based, two-dimensional, proton-detected (H)COH spectra with varying CP contact times were recorded at 60 kHz MAS and an actual sample temperature of 291 K. The applied transfer time for both CP steps (^{1}H-^{13}CO and ^{13}CO-^{1}H) is indicated in each panel that show the eight experiments. The 200 µs-experiment (top left) is plotted with contours at the noise level, while all other spectra are plotted at the same contour levels (positive contours are shown in black and red for the D96-$H^{\delta2}/C^{\gamma}$ and D85-$H^{\delta2}/C^{\gamma}$ cross peaks, respectively, and negative contours are shown in grey).

The build-up of the two cross peak intensities was analyzed quantitatively (bottom panel, the D96-$H^{\delta2}/C^{\gamma}$ and D85-$H^{\delta2}/C^{\gamma}$ data points are shown in black circles and red squares, respectively). We fitted the two intensities over the CP contact time with equation (1) using IGOR Pro Version 8.03 and the nonlinear least square method (shown in dashed lines):

$$\mathrm{I(t)} = \mathrm{I_{max}}(1 - \mathrm{e}^{-kt}); \quad k = \frac{1}{\mathrm{T_{HC}}} \tag{1}$$

with the CP contact time t, cross peak intensity I, and the CP build-up time T_{HC} of the cross peak (k is the build-up rate). The obtained coefficient values ± one standard deviation are given in the plot for both T_{HC} and I_{max} for the D96-$H^{\delta2}/C^{\gamma}$ and D85-$H^{\delta2}/C^{\gamma}$ cross peaks. The D96-$H^{\delta2}/C^{\gamma}$ cross peak (T_{HC} = 310.79 µs) builds up faster by a factor of about 2.3 than the D85-$H^{\delta2}/C^{\gamma}$ signal (T_{HC} = 692.71 µs). Assuming that deuteration effects and ^{13}C-^{1}H dipolar couplings, i.e. dynamics of the D96 and D85 carboxyl groups, are similar, this indicates that, comparing D96 and D85, the carboxylic acid proton is closer to the C^{γ} in the case of D96. However, potentially different side chain mobilities of the D85 and D96 residues may additionally contribute to the observed differences in CP build-up.

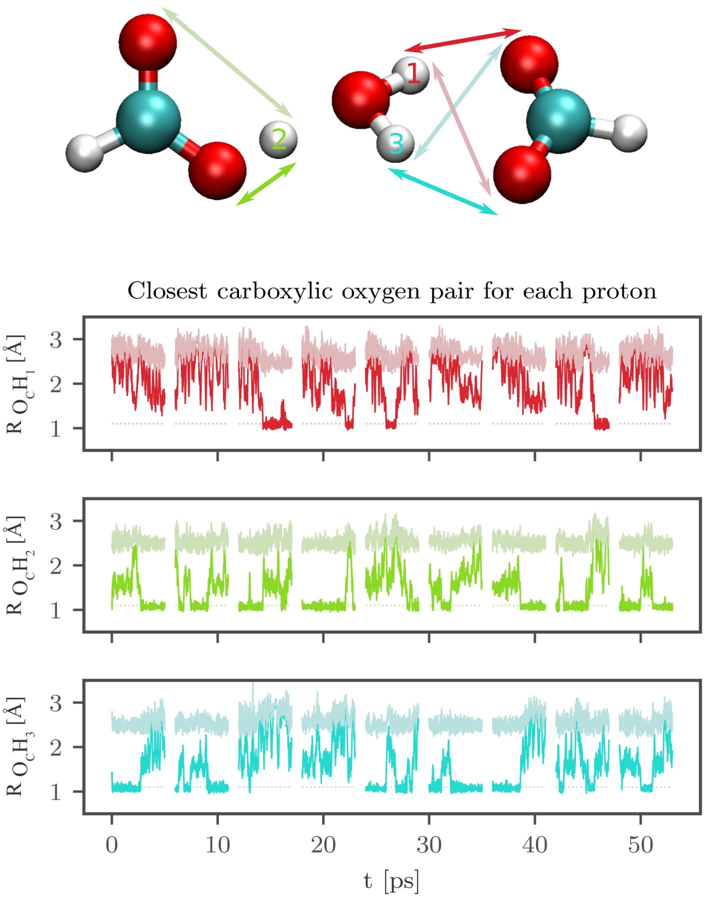

Supplementary Figure 6. Analysis of occurring proton-oxygen distances between H_2O and carboxyl groups in *ab initio* molecular dynamics simulations. The model system used in this study consists of two carboxyl groups, one H_2O molecule and one excess proton (see also Fig. 3b in the main text). For each of the three protons (red, green and cyan), the shortest proton-oxygen distance (plotted in bright colors) is computed for each of the nine NVE trajectories and compared to the distance of the same proton to the other oxygen (plotted in muted colors) in the respective carboxyl group. The distances among all protons vary strongly, providing evidence that bifurcated, i.e. three-centered, hydrogen bonds are not observed in the model system during the molecular dynamics simulations.

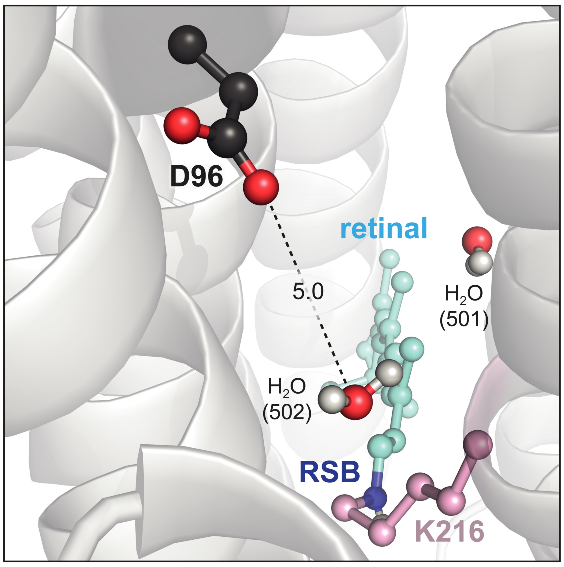

Supplementary Figure 7. Structural view of the bacteriorhodopsin entry site in the dark-state crystal structure (pdb 1c3w). At the intracellular proton uptake site, the D96 carboxyl group and water molecule 502 are 5.0 Å from each other (indicated by the dashed line).

Supplementary Table 1. Observed chemical shifts of backbone amides in bacteriorhodopsin. Signals have been assigned through sequential connections employing triple-resonance magic angle spinning NMR experiments (all values are given in p.p.m.).

	$^{1}H^{N}$	^{15}N
**A39**	8.03	123.75
**K40**	8.52	116.11
**K41**	7.29	117.73
**F42**	7.15	115.03
**Y43**	8.88	120.67
**A44**	8.18	123.61
**I45**	7.76	114.42
**F71**	7.58	114.01
**G72**	9.52	109.69
**G73**	8.55	105.85
**E74**	7.15	117.72
**Q75**	8.43	118.83
**N76**	7.78	122.48
**I78**	8.75	125.88
**Y79**	8.45	127.51
**W80**	7.32	125.03
**A81**	6.26	124.23
**G106**	8.93	104.83
**T107**	7.34	118.08
**I108**	7.70	119.36
**L109**	8.05	118.66
**A110**	7.71	118.65
**T128**	7.30	118.23
**K129**	8.84	118.80
**V130**	7.75	117.19
**Y131**	8.74	128.38
**S132**	8.70	109.94
**E166**	9.75	116.24
**V167**	7.30	120.50
**A168**	7.65	121.12
**S169**	8.36	110.87
**T170**	7.23	118.66
**G195**	6.76	105.91
**A196**	9.53	129.23
**G197**	8.28	106.46
**I203**	6.73	118.98
**E204**	8.59	120.94
**T205**	8.31	113.20
**L206**	7.07	126.45
**L207**	7.55	121.23
**F208**	9.23	125.72

Supplementary Table 2. Observed chemical shifts of key sites involved in the proton transport pathway of bacteriorhodopsin (BR). All chemical shifts are observed by direct ^{1}H detection and are further discussed in the main text. The retinal Schiff base (RSB) ^{15}N and ^{1}H chemical shifts have been obtained at 97 K, whereas the R82, D96 and D85 chemical shifts are observed by measurements at around 290 K. All values are given in p.p.m. and apply to BR dark-state.

	^{1}H / $^{1}H^{N}$	$^{1}H\eta$	$^{1}H\delta 2$	$^{13}C\gamma$	^{15}N	$^{15}N\eta$
**R82**	–	6.24	–	–	–	77.65
**D85**	–	–	12.12	169.68	–	–
**D96**	–	–	11.04	170.78	–	–
**RSB (BR_{555})**	13.22	–	–	–	173.51	–
**RSB (BR_{568})**	12.24	–	–	–	165.42	–
